# Chilean Papaya (*Vasconcellea pubescens*): A Native Fruit with a High Health-Promoting Functional Potential

**DOI:** 10.3390/antiox13121521

**Published:** 2024-12-12

**Authors:** Roberto Lemus-Mondaca, Luis Puente-Díaz, Angélica Cifuentes, Katherine Lizama, Paula González

**Affiliations:** 1 Department of Food Science and Chemical Technology, Faculty of Chemical Sciences and Pharmaceutical, Universidad de Chile, St. Dr. Carlos Lorca 964, Independencia, Santiago 8380494, Chile; angelica.cifuentes@ug.uchile.cl (A.C.); katherine.lizama@ug.uchile.cl (K.L.); paula.gonzalez.3@ug.uchile.cl (P.G.); 2 Department of Agricultural, Food & Nutritional Science, University of Alberta, Edmonton, AB T6G 2P5, Canada

**Keywords:** Chilean papaya, *Vasconcellea pubescens*, nutritional components, antioxidant, papaya latex

## Abstract

Papaya fruit is commonly known for its nutritional and medicinal value. It is a perennial, herbaceous, and trioecious cross-pollinated species with male, female, and hermaphrodite plants. The Chilean papaya, originating from South America, has been extensively spread throughout the Andean nations, cultivated primarily in the Coquimbo and Valparaíso valleys in Chile, between 34°41′ and 36°33′ latitude south. Its intense aroma, yellow color, and oblong shape characterize this fruit. It also stands out for its high content of carotenoids, vitamins, proteins, and polysaccharides, which make it a great functional food. Also, papaya contains bioactive compounds with antifungal, anti-inflammatory, and wound-healing effects. For years, the fruit has been used to produce canned fruit, juice, and candies to satisfy the local market. Chilean papaya has significant economic importance, supplying both local and international markets. This review aims to consolidate the evidence-based information on the native Chilean papaya (*Vasconcellea pubescens*) as a food matrix. The fruit’s ripening process, nutritional composition, industrial applications, and health-promoting properties, including its antioxidant and antidiabetic effects, are thoroughly examined. Additionally, the extraction of papaya oil, encapsulation of bioactive compounds, industrial and artisanal processing techniques, and patents are explored, highlighting the diverse applications and potential benefits of this fruit.

## 1. Introduction

**Figure 1:**
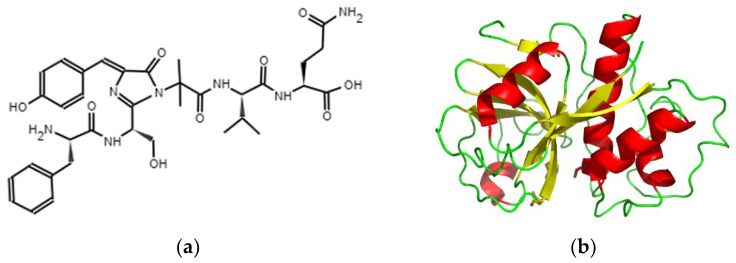
Papain’s chemical structure (**a**), Caricaceae’s 3D papain structure (**b**) [5].

The Chilean papaya is a native fruit of South America that has been widely distributed in the Andean countries. It belongs to the *Caricaceae* family and corresponds to *Vasconcellea pubescens*. This species was introduced in Chile more than 50 years ago and is cultivated in the Coquimbo and Valparaíso valleys, as well as on the coast of the Maule Region [1] between 34°41′ and 36°33′ latitude south. This fruit is characterized by its intense aroma, yellow color, and oblong shape. In addition, ripe papaya is an excellent source of carotenoids, vitamins, proteins, and polysaccharides [1]. Papaya is an essential fruit in Chile and has aroused great interest due to its wide use in preparing different food products, such as papaya gummies, canned papaya, juice, syrup, and jam [2], besides having antifungal properties, anti-inflammatory effects and wound-healing effects [3]. Additionally, this species produces latex with a high level of papain (Figure 1), an important and valuable proteolytic enzyme that plays a fundamental role in the digestive process by participating in the breakdown of strong protein fibers [4].

Papain is a cysteine protease isolated from papaya latex, a milky exudate that protects green fruits and young leaves. Structurally, papain is a globular protein containing around 212 amino acids with an approximate molecular weight of 23,406 Da. Its three-dimensional structure includes an alpha/beta domain, with a central beta sheet surrounded by alpha helices. The active site of papain consists of a catalytic triad formed by cysteine, histidine, and asparagine residues, which are crucial for its enzymatic activity. The greener the fruit, the more active the papain. This enzyme exhibits high activity in the hydrolysis of proteins, oligopeptides, amino acid esters, particularly in arginine, lysine, residues following phenylalanine, and amide bonds in general [5,6]. It is stable and active under various conditions, even at elevated temperatures. The protein is stabilized by three disulfide bridges, along which the molecule folds, creating strong interactions among the side chains, which contribute to the stability of the enzyme [6].

It is a component widely used in different medicinal lines, cell isolation, detergents, leather and textiles, cosmetics, the pharmaceutical industry, and dermatological products, and in foods mainly as a beer clarifier and meat tenderizer [7]. 

Only 225 hectares of *Vasconcellea pubescens* are cultivated in Chile, mainly concentrated between 30° and 33° latitude south (94%). Most orchards in this area are relatively large (10–40 ha) and maintained under standard fruit management practices (terracing, fertigation, pest management, pruning, etc.), supplying local and international markets. This species is also cultivated in southern Chile (35°–38° South Latitude) but in smaller orchards, primarily in home gardens [8]. Its life cycle begins after a brief fall-winter dormancy between June and August, when the average temperature exceeds >20 °C. New leaves, flowers, and fruits begin to grow in spring, with fruits from the previous season also starting to mature. This process continues annually between September and May [9].

The objective of this review is to highlight the potential of Chilean papaya by detailing its origin, nutritional characteristics, medicinal properties, industrial applications, and life cycle. This includes its role as a source of bioactive compounds (such as papain) and its multiple uses in food, cosmetics, and medicine, as well as its relevance in local and international markets and its potential in research.

## 2. Papaya Fruits Varieties

Papaya fruits grow in tropical and subtropical regions and are traded worldwide. They can be found in valleys at elevations up to 2000 m above sea level. Numerous studies have described the beneficial effects of this fruit against chronic diseases such as cancer, diabetes, and obesity [10]. 

The *Caricaceae* are a small family of six genera and 35 species (Table 1), and they have a disjunct distribution between Africa and the Neotropics. *Cylicomorpha*, the only African genus of *Caricaceae*, has two large tree species restricted to humid premontane forests in East and West Africa. The other five genera occur in South and Central America with representatives in wet and seasonally dry habitats. *Vasconcellea*, the largest genus within the family, comprises 20 species plus a naturally occurring hybrid, *Vasconcellea* × *heilbornii*. Due to its prominent economic importance, the unique species of *Carica*, the common papaya (*C. papaya* L.), has become pantropical [11]. The second-largest genus is *Jacaratia*, with seven species of trees; it is widespread in the lowlands of the Neotropics. *Horovitzia* and *Jarilla* are mainly herbaceous plants of tropical seasonal forests in Mexico. *Horovitzia cnidoscoloides*, the only species in the genus *Horovitzia*, is a small, thin tree. It is monotypic and endemic to the Sierra de Juárez in northern Oaxaca, Mexico. *Jarilla* comprises three species of herbs with perennial tubers that resprout and can be found in Guatemala.

## 3. Chilean Papayas

Chile is characterized as a long and narrow country with tremendous climatic variation, which makes it a suitable climate for papaya cultivation in certain regions. For this reason, in Chile, there are two varieties of papaya. One of these varieties is the introduced papaya (*Vasconcellea pubescens*), and the other is the Chilean origin papaya (*Vasconcellea chilensis*) [19]. It is still being determined if *Vasconcellea chilensis* was an evolution because of human selection or if it was present in the wild ancestors. Still, they also claim this variation has valuable introgression characteristics concerning other papaya species [19,20]. During this period, there was a lack of clarity regarding *Vasconcellea pubescens*, which was often used synonymously with the genus *Carica* (synonyms of *Vasconcellea cundinamarcensis*, *Carica pubescens*, initially called *Carica candamarcensis*).

**Figure 2:**
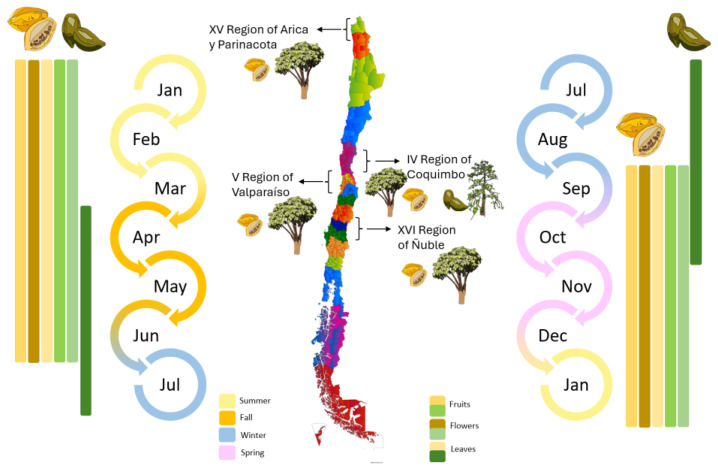
Regional map of Chile showing papaya cultivation by geographical zone and life cycle of *Vasconcellea pubescens* (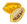
—orange palette) and *Vasconcellea chilensis* (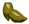
—green palette) [9,23,25].

The literature describes this confusion between the two genera due to their resemblance and typical ecological preference for higher altitudes. It is understood that there are differences in climate conditions, peak production months, and genetic evidence distinguishing them [21,22]. The Natural Resources Research Center, CIREN [23] reports that the regions of Arica and Parinacota, Coquimbo, Valparaíso, and Ñuble (Figure 2) have hectares of papaya plantations with 135.77 h total. The region of Coquimbo produces 83.81% of the area of papaya in Chile, where 100% of papaya production is destined for the agro-industry [24].

### 3.1. Vasconcellea pubescens

Papaya (*Vasconcellea pubescens*) is a tropical species cultivated in Chile. It is assumed to be a trioecious plant [8] and is described as dioecious (with male and female plants) and monoecious, featuring scarce bisexual flowers that produce deformed fruits [26]. It reaches a height of 3–6 m, sometimes up to 10 m [27], and has succulent, medullose, and branched stems with an extensive basal part [28]. The fruit of *Vasconcellea pubescens* is yellow and obovoid or ellipsoidal, five-sided, 6–15 cm long, and 3–8 cm wide [29]. It is essential to mention that the edible yield is only 46% of the whole fruit, with approximately 5% sugar content [30]. Its taxonomy comes from the Plantae Kingdom, *Brassicales* Order, *Caricaceae* Family, *Vasconcellea* gender, *Vasconcellea pubescens* species. *Vasconcellea pubescens* is cultivated in several South American countries [25]. In Chile, it is grown from 29°54′ S–71°15′ W (La Serena city) to 36°08′ S–72°47′ W (Cobquecura city), always under moderate temperatures (10–25 °C) because it cannot tolerate frost or temperatures over 30 °C [9]. The timing of its introduction to northern Chile is still not precise, but the first Spanish chronicles indicate that native people had already cultivated it before the Spanish conquerors arrived in this region around 1535. [31]. *V. pubescens* is considered a food heritage site in the area and the country.

### 3.2. Vasconcellea chilensis

*Vasconcellea chilensis* is harvested from dioecious papaya, and has a branched succulent stem that is cylindrical, short, and thick, 1 to 4 m high, with flowers of 1–3 cm diameter. Its taxonomy comes from the Plantae Kingdom, *Magnoliophyta* Division, *Magnoliopsida* Class, *Brassicales* Orden, *Caricaceae* Family, and *Vasconcellea* Genus [32]. The fruit it produces measures from 1 and 2 to 3 cm and has a greenish-brown color, much smaller than the papaya (*Vasconcellea pubescens*) (Table 2) [19,33]. Its current fragmented natural populations are between 29°25′ S–71°17′ W and 30°42′ S –71°22′ W (Coquimbo, Chile) [25]. This papaya species is estimated to be in danger of extinction by the extreme that small populations meet and the destruction of its original habitat [19]. That is why it is estimated that they are not registered explicitly within Chile’s harvested fruit statistics.

## 4. Ripening and Shelf Life

The fruit has a strong and characteristic aroma. The ripe fruit is used to make preserves and drinks. *Vasconcellea pubescens* fruit has been classified as a climacteric fruit [22]. Climacteric fruits exhibit a characteristic rise in ethylene production and respiration during ripening [43,44]. Its life cycle begins after emerging from a brief fall-winter break between June and August (Figure 2) [9]. The first noticeable event during the ripening of *Vasconcellea pubescens* is the rapid degreening of the skin, which is followed by the climacteric ethylene rise, where the ethylene production reached values of 44 μL kg^−1^ h^−1^ after 11 days of storage at 20 °C [45]. In addition, a rapid softening of the flesh is apparent. Ethylene plays a key role during ripening, stimulating the development of ripening attributes such as color, texture, aroma, and flavor [22].

As the ripening develops, a slow increase in pH and a reduction in titratable acidity are observed during storage at 20 °C. While dramatic changes in skin color occur in the first 3 days of storage, a marked increase in total soluble solids occurs, and after that, a drop is observed [22].

**Figure 3:**
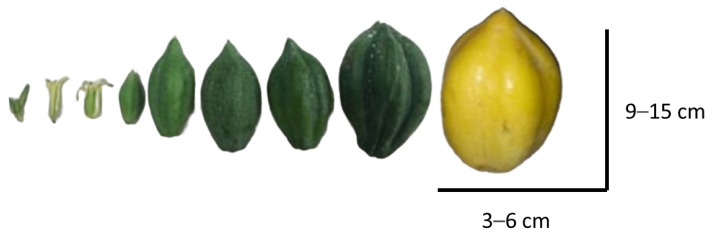
Evolution of the *Vasconcellea pubescens* fruit (unripe green fruits and ripe yellow fruit (180–200 g)) [9].

The rate of degreening is not homogenous among different fruits. For almost total degreening (90% yellow), between 3 and 5 days at 20 °C are required, starting from green to 10% yellow fruit, as shown in Figure 3. This degreening rate is faster than reported for different Chilean papaya cultivars and related species, such as ‘Sunrise’ and ‘Kapolo’ [46].

In the research by Caicedo Pineda and García Ortiz (2018), titled “Evaluation of Physicochemical Parameters During the Growth, Development, and Postharvest of the Species *Vasconcellea pubescens*, *Solanum quitoense* var. *septentrionale*, and *Capsicum pubescens*”, the polar diameter (length) and axial diameter (width) at different maturity stages was measured. They developed a growth curve for the species *Vasconcellea pubescens* during the development cycle over 22 days of evaluation and determined that both the diameter and length of the fruit increased proportionally, as shown in Figure 3 [47].

## 5. Nutritional Components

Papaya contains a critical number of vitamins (A, B_1_, B_2_, and C), minerals (calcium, iron, potassium, and sodium), and carotenoids (lycopene, β-carotene, and β-cryptoxanthin) while being low in sodium, fats, and calories. In addition to its striking aroma and high vitamin content, the fruit of *Vasconcellea pubescens* is attractive to consumers because it contains numerous phenolic compounds, specifically hydroxycinnamic acid and quercetin glycoside derivatives, which play an essential role in the anti-hyperglycemic, antioxidant, and insulin stimulating activities [48].

It has been determined that the nutritional properties expressed in Table 3 are important because they help to improve health and digestion, reduce harmful cholesterol levels, and prevent diabetes, among other benefits. On the other hand, it also highlights the qualities of latex for healing and protection against the development of gastric ulcers. In addition, oleic acid predominates in its seeds and benefits the blood vessels, reducing the risk of cardiovascular diseases [49]. In addition, papaya has a low content of lipids and proteins. However, it has a high moisture content; total carbohydrates are obtained by difference. The papaya fruit is generally rich in bioactive compounds such as phenolic compounds and vitamin C, contributing to antioxidant capacity [1].

## 6. Bioactive Compounds

Phenolic compounds are specialized metabolites primarily found in plant species, characterized by significant structural diversity. They are known for their antioxidant potential, which can prevent or repair damage caused by oxidation [52,53]. The most abundant antioxidants in fruits are polyphenols and vitamin C, while vitamins A, B, and E, and carotenoids, are present in lesser quantities in some fruits [54]. Moreover, papaya contains a significant concentration of carotenoids, vitamins, proteins, and polysaccharides [1].

Diverse studies have identified bioactive compounds in Chilean papaya following distinct processing methods and different parts that comprise the fruit (Table 4). Vega-Gálvez et al. (2021) [55] compare the bioactive components of fresh papaya with papaya subjected to various drying processes. Among the best results, vacuum-drying papaya maintains 5.83 (mg GAE g^−1^ D.M.) of total phenolic content and 2.50 (mg QE g^−1^ D.M.) of total flavonoid content, freeze-drying papaya maintains 21.16 (μmol TE g^−1^ D.M.), and infrared drying papaya maintains 66.83 (μmol TE g^−1^ D.M.). Conversely, an additional study using the same approach identified other compounds of interest in the peeled papaya, obtaining the best results of gallic acid 9.76 ± 0.61 (mg 100 g^−1^ D.M.), chlorogenic acid 4.38  ±  0.49 (mg 100 g^−1^ D.M.), Tyrosol 21.01  ±  0.74 (mg 100 g^−1^ D.M.), and naringin 3.34  ±  0.12 (mg 100 g^−1^ D.M.) in vacuum drying, and ρ-Coumaric acid 7.84  ±  0.12 (mg 100 g^−1^ D.M.), and trans-ferulic acid 5.56  ±  0.06 (mg 100 g^−1^ D.M.) in infrared drying [48]. Uribe et al. (2015) [1] determined extraction by agitation, ultrasound, high hydrostatic pressure, and a combination of them like high hydrostatic pressure-agitation, and high hydrostatic pressure-ultrasound extractions (HHPE-UE), HHPE-UE was the most considerable extractive process obtained from bioactive compounds. Briones-Labarca et al. (2015) [2] determined the bioactive compounds present in the seeds of the Chilean papaya by high hydrostatic pressure extraction (HHPE) and ultrasound-assisted extraction to different time processes. Then, 15 min for HHPE is the best process extraction, but 5 min for HHPE processes is more acceptable energetically. 

On the other hand, it is interesting to estimate the bioactive compounds present in another component of the fruit plant. Halder et al. (2022) [56] studied the bioactive compounds present in the flower of the *Carica* papaya, where they obtained values of 108.66 ± 0.35 phenols (mg GAE 100 g^−1^), 13.7 ± 0.57 flavonoids (mg QE 100 g^−1^), 260.83 ± 0.57 CUPRAC (mg GAE g^−1^), and 59.33 ± 0.52 FRAP (µMFe^2+^ g^−1^), results that could be intriguing to investigate further in the plant of Chilean papaya. On the other hand, Robles-Apodaca et al. (2024) [57] successfully optimized the extraction process of bioactive compounds from papaya seeds using response surface methodology (RSM) combined with a central composite design (CCD). This approach maximized the total yield of polyphenols and flavonoids, and antioxidant capacity, as assessed through ABTS and DPPH assays.

**Table 4:** Total phenolic, antioxidant activity, and other bioactive compounds of Chilean papaya (method with the best result).

Reference	[55]	[48]	[1]	[2]	[58]	
Extraction	Pulp with Skin	Pulp	Pulp with Skin	Seeds	Mucilage and Seeds	
	Fresh	Fresh	HHPE-UE	HHPE 15 min	^‡^ PM	^†^ PEA
Total phenolic content	7.29 ± 0.40 mg GAE g^−1^ D.M.	7.02 ± 0.42 mg GAE g^−1^ D.M.	129.1 ± 3.8 mg GAE 100 g^−1^	<7 mg AG g^−1^ seed	4.902 ± 0.702 g GAE 100 g^−1^ of extract	6.244 ± 0.342 g GAE 100 g^−1^ of extract
Total flavonoid content	2.51 ± 0.25 mg QE g^−1^ D.M.	3.33 ± 0.26 mg QE g^−1^ D.M.	N/A	<2.5 mg quercetin g^−1^ seed	N/A	N/A
Sulforaphane	N/A	N/A	N/A	54.97 ± 0.90 mg g^−1^ seed	N/A	N/A
DPPH ^2^	39.07 ± 5.68 μmol TE g^−1^ D.M.	81.26 ± 1.23 µmol TE g^−1^ D.M.	20.6 ± 0.2 mg TE g^−1^	<110 μmol TE g^−1^ seed	94.80 ± 2.69 μg mL^−1^	55.99 ± 3.55 μg mL^−1^
FRAP ^3^	N/A	N/A	97.2 ± 4.3 mg TE g^−1^	<115 μmol TE g^−1^ seed	N/A	N/A
ORAC ^4^	107.2 ± 5.17 μmol TE g^−1^ D.M.	55.20 ± 0.58 µmol TE g^−1^ D.M.	N/A	N/A	N/A	N/A
Voltammetry	N/A	N/A	141.0 ± 13.8 mM TE 100 g^−1^	N/A	N/A	N/A
Phenolic						
Caffeic acid	N/A	N/A	1.5 ± 0.1 mg 100 g^−1^	N/A	N/A	N/A
Trans-Ferulic acid	N/A	N/A	0.86 ± 0.1 mg 100 g^−1^	N/A	N/A	N/A
Rutin	N/A	N/A	2.8 ± 0.3 mg 100 g^−1^	N/A	N/A	N/A
Other						
Vitamin C	N/A	7.27 ± 0.11 mg g^−1^ D.M	74.1 mg 100 g^−1^ FW	N/A	N/A	N/A
β-carotene	N/A	2595 ± 65.0 µg 100 g^−1^ D.M.	N/A	N/A	N/A	N/A
Gallic acid	N/A	2.41 ± 0.30 mg 100 g^−1^ D.M.	N/A	N/A	N/A	N/A

N/A: Not applicable, ^2^ DPPH: 2,2-diphenyl-1-picrylhydrazyl, ^3^ FRAP: ferric reducing antioxidant power, ^4^ ORAC: radical absorbance capacity, ^‡^ PM: papaya methanol extract, ^†^ PEA: papaya ethyl acetate extract. Vega-Gálvez et al. (2021) [55]; Vega-Gálvez et al. (2019) [48]; Uribe et al. (2015) [1]; Briones-Labarca et al. (2014) [2]; Pino-Ramos et al. (2024) [58].

## 7. Papaya Latex

Latex corresponds to cytoplasm present in species that have a structure called laticifers. This cell forms a tubular network where the latex circulates, so a cross-section of the cell tissue will give off a milky substance known as latex [59]. Papaya latex is usually extracted from the immature fruit of green color and can be enveloped by various environmental and physiological variables [60]. According to Chen et al. (2012) [59], papaya latex contains 15% solid matter, of which 40% corresponds to enzymes such as cysteine endopeptidases (such as papain, glycyl endopeptidase, chymopapain, and caricain make up 80% of all enzymes), chitinolytic enzymes, glycosidase, glutaminyl cyclotransferase, and peroxidase, among others. Papain was the first cysteine to be characterized as a latex component of papaya fruit and is known as a 14 N-terminal structure of proteinases from the *Caricaceae* species [61,62,63].

It is essential to perform a thermal process on the enzyme papain before consumption. This process is characterized by scalding the tongue, reddening the area, and sensitizing it. However, this does not dismiss the multiple health properties attributed to it.

A study by Jiménez et al. (2003) [64] on Chilean papaya (*V. pubescens*) determined no statistical difference in the specific activity of latex extracted in different seasons of the year. Vidal et al. (2009) [60] performed the same experiment, finding significant differences only when analyzing different sizes of the green fruit in the other seasons, establishing that there is a higher yield in spring associated with an activity complementary to the production of the fruit along with an increase in height and diameter of the trunk of the plant that exerts the same effect. On the other hand, there is almost zero yield in autumn because there are no green fruits in that season.

Several studies detail that the method used to collect papaya enzymes is drying (Table 5), obtaining a dried power product between a white and brown color, to which different names will be attributed depending on the degree of purity obtained [60]. Jiménez et al. (2003) [64] determined a lower papaya color alteration in a freeze-drying process than in conventional or vacuum drying.

The papain enzyme is a protein with a molecular weight of 24,500 Da and a polypeptide containing three disulfide bridges and a sulfhydryl group that is required for the enzyme’s activity [6].

The active site of the enzyme is located in a cleft between two unique structural domains in the three-dimensional structure [66].

The Cys-25 region of the active site, which attacks the carbonyl carbon in the peptide chain’s backbone to release the amino-terminal region and causes the protein to disassemble, enables Papain’s method of action [6].

Hydrophobic interactions influence the conformation of the protein and, hence, its functionality. The hydrophobic amino acids alanine, leucine, methionine, valine, and isoleucine are responsible for the hydrophobicity of the papain enzyme. The hydrophobicity of amino acids within a protein’s secondary sequence significantly determines its folding behavior and structural arrangement. The enzyme’s hydrophobic core plays a crucial role in stabilizing its tertiary structure through intramolecular interactions, while the polar regions on its surface ensure compatibility with the surrounding aqueous environment, maintaining both structural integrity and functionality [66].

The study [65] characterized the latex of papaya from three species of the genus *Vasconcellea*. It was observed that *V. pubescens* had a total latex value of 214.71 g, compared to *V. x heilbornii* and *V. chachapoyensis*, which exhibited values of 167.64 g and 334.32 g, respectively. Regarding enzymatic activity, *V. pubescens* showed an average value of 195.8 Upe, while *V. x heilbornii* and *V. chachapoyensis* demonstrated values of 112.45 Upe and 240.97 Upe, respectively, at pH 6.0.

The differences in latex value likely reflect inherent genetic and biochemical variations in the case of enzymatic activity, indicating variations in hydrophobic interactions and folding of the papain enzyme.

Papaya enzymes are highly studied and used in different areas such as food and pharmaceuticals, either to accelerate industrial processes, for their biological functions or their nutraceutical and antimicrobial properties, for their antidiabetic activity, and anticancer and antioxidant properties, among others reported by Ghaffar et al. (2023) [67].

## 8. Antidiabetic Potential

The antihyperglycemic effect of papaya is believed to target pancreatic beta cells by increasing their sensitivity to insulin while simultaneously inhibiting α-amylase and α-glucosidase. The contribution of flavonoids, tannins, and alkaloids in various parts of the papaya is also suspected to play a significant role [68].

The α-Glucosidase inhibitors are therapeutic agents for the treatment of type 2 diabetes. Acarbose and miglitol are notable FDA-approved drugs. However, their regular use may lead to various side effects, including diarrhea, vomiting, flatulence, severe abdominal pain, allergic reactions, and others [69,70].

α-Glucosidase is composed of 811 amino acids with a structural architecture comprising 35% helices, 25% β-sheets, and 38% coils. It exhibits a resolution of 2.04 Å, as determined through X-ray diffraction studies. Ramachandran plots indicated that 97.6% of the residues are in favored regions, demonstrating high precision in the phi (φ) and psi (ψ) angles of the target protein’s coordinates [71].

The enzyme α-glucosidase catalyzes the final step in carbohydrate digestion and breakdown, making it a critical target for studies investigating its inhibition through the polyphenolic composition of various fruits. These studies often correlate antioxidant capacity with antidiabetic activity [40,66,72]. This behavior is associated with the chemical structure of the compounds, particularly the presence of a methoxy group with electron-donating and electron-withdrawing properties [73].

**Figure 4:**
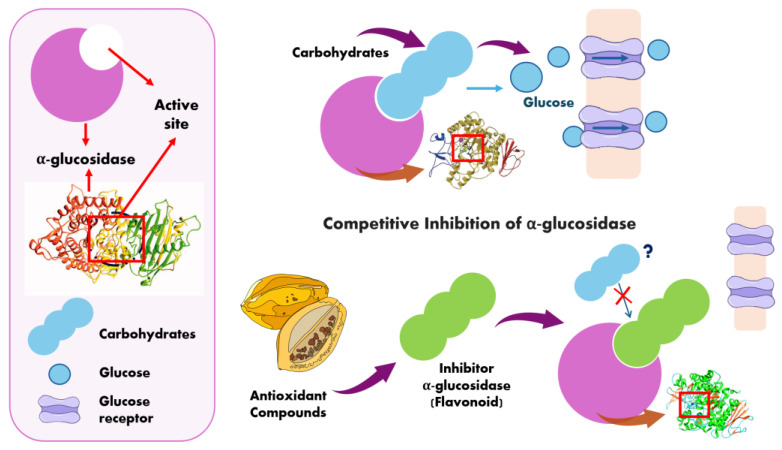
Mechanism of competitive inhibition of α-Glucosidase [71,74].

Due to their structural similarity to carbohydrates, such as disaccharides or oligosaccharides, α-glucosidase inhibitors can form high-affinity complexes with the enzyme’s active site through competitive inhibition (Figure 4). This mechanism reduces the formation of carbon monomers and glucose absorption [70,74].

Carbohydrates that are not hydrolyzed become undigested carbohydrates and are transported to the large intestine, where they are fermented into short-chain fatty acids or gases. Alternatively, they may act as prebiotics or simply reach the end of the digestive process as feces [75,76,77].

Vega-Gálvez et al. (2019) [48] studied α-glucosidase activity in Chilean papaya and the effects of different thermal preservation treatments on the fruit. The study determined that fresh papaya initially had an IC_50_ of 312 mg/mL. Notably, the sample treated with infrared drying was the most effective in inhibiting α-glucosidase, 24 times more effective than the fresh sample.

In a comparative analysis of Chilean Papaya (*Vasconcellea pubescens*) with the variety *Carica papaya* L., a clear difference in α-glucosidase IC_50_ values is observed (Table 6), which appears to be directly related to the total polyphenolic content (TPC) of the fruits [10,48,78]. Similarly, a study by Mohamed et al. (2024) [79] explored, through in vitro and in silico methods, the inhibitory effects of *N. Latifolia* fruit aqueous extract on carbohydrate-digesting enzymes. The study revealed the low fluctuation, high stability, and strong binding affinity of these enzymes with the compounds, as demonstrated by molecular dynamics simulations. These findings suggest promising inhibitory results compared to the natural inhibitor acarbose.

Although promising results have been reported on the α-glucosidase inhibitory effects of fruits such as Chilean papaya, further in vivo investigations are essential to confirm these findings and establish their potential as bioactive agents for preventing and treating type II diabetes. These inhibitors improve the metabolic profile and may potentially reduce the risk of long-term complications associated with hyperglycemia.

## 9. Papaya Oil

Around 13.9–30.7% of the fruit’s volume is comprised of seeds, which are abundant in protein, lipids, crude fiber, monounsaturated fatty acids, and various functional constituents, such as carotenoids, polyphenols, prunasin, benzyl glucosinolates, benzyl isothiocyanates (BITC), and cyanogenic substances [80,81]

**Figure 5:**
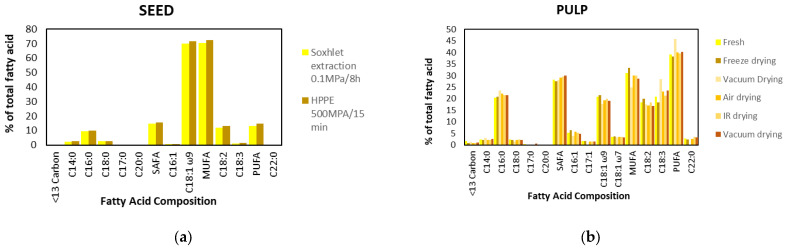
Oil Chilean papaya (*V. pubescens*) fatty acid composition (% of total fatty acid), seed (**a**) [2] and pulp (**b**) [84].

The oil from Chilean papaya (*V. pubescens*) is extracted from its seeds. It is currently sold in “Elquimia”, a small shop, as a skin care product, attributing several antioxidant properties, essential fatty acids, and the small concentration of enzymes typical of papaya. Krist (2020) [82] indicates that the *Carica papaya* is 28% seed oil, 54% endosperm oil, and 7% sarcotesta oil. These percentages correlate with the information extracted from Figure 5, which indicates the lipid composition of the seeds and papaya pulp (*V. pubescens*) in different extraction and drying treatments.

The extraction method can significantly affect yield, quality, and antioxidant activity. Typically, oils are extracted through mechanical pressing and organic solvent extraction methods [83]. However, studies also use extraction methods for the extraction of supercritical fluid, and extraction assisted with high hydrostatic pressure (HHPE) or ultrasound, as well as the influence of drying pretreatments, to extract oil from the seed or pulp of the papayas (Figure 5) [2,84].

Although there is no documented evidence regarding the oxidative stability of Chilean papaya oil, a study indicates that papaya oil from the species *Carica papaya* L. contains 71.30% oleic acid with an oxidative stability of 77.97 h [85].

The fatty acid composition of Chilean papaya oil extracted from its seeds makes it comparable to well-known market and industrial oils, particularly due to its high oleic acid content, which exceeds 70% of the total. This is similar to olive oil, which has an oleic acid content of 70% [2,86] and FRAP antioxidant capacities of 120 (μmol TE g^−1^) and 107 (μmol TE g^−1^) for papaya and olive oil, respectively [2,87] Olive oil is known for its nutritional, health-promoting, functional, emulsifying, and antioxidant properties [88]. A study by Lu et al. (2023) [89] highlights the protective effects of oleic acid against cardiovascular diseases through multiple cellular mechanisms and signaling pathways, based on preclinical and clinical research. This provides a promising perspective on the potential properties that Chilean papaya oil may exhibit.

## 10. Encapsulation of Chilean Papaya Compounds

Cañas-Sarazúa et al. (2024) [83] studied the effect of the encapsulation of Chilean papaya seed oil with alginate casein shell. They obtained microcapsules of high mechanical stability, demonstrating the protective effect obtained from components of microcapsules in gastric conditions, with a release of seed oil in the intestinal phase at 52.23%, and a bioaccessibility of 75.36%.

Fuentes et al. (2023) [90] investigated the microencapsulation of bioactive extracts from the seeds and skin of the Chilean papaya, which showed antimicrobial and antioxidant properties. Among the best results obtained were in the extract of microencapsulated seed with maltodextrin at 20%, reporting 44.20 ± 3.32 EAG g^−1^ for total phenols with an antioxidant capacity of 12.0 ± 0.32 mol ET g^−1^. In the case of the seed samples, the antimicrobial potential was particularly effective against *Staphylococcus aureus*, with a minimal inhibitory concentration of 0.03 mg mL^−1^ for the extract and 0.25 mg mL^−1^ for microencapsulation using all concentrations of maltodextrin.

## 11. Chilean Papaya Processing

Researchers have studied the conditions of various traditional preservation treatments in Chilean papaya, identifying the bioactive compounds of interest (Table 7). Additionally, they have developed mathematical models that best fit drying treatments under different parameters, moisture isotherms, and rehydration capacity [30,91]. They also determined how vacuum treatments enhance mass transfer, irrespective of the °Brix concentration of a solution [92].

## 12. Industrial and Craft Technologies

The papaya contains approximately between 7 and 9% of total sugars. It is mainly consumed as fresh fruit, for dessert or salad. There are flavor variations when they ripen in the summer months, since their sugar content is higher. The seeds have a pungent taste. Traditionally, the ways to industrialize papaya have been canning (fruit juice), juice and nectar, honey, jam, and candied fruit. Preferably, the mesocarp (pulp) of the fruit is processed, and in very few cases, the rest is taken as a surplus of the total industrialized volume; between 50 and 60% is done at an artisanal level with very elementary techniques and rudimentary infrastructure [94].

**Figure 6:**
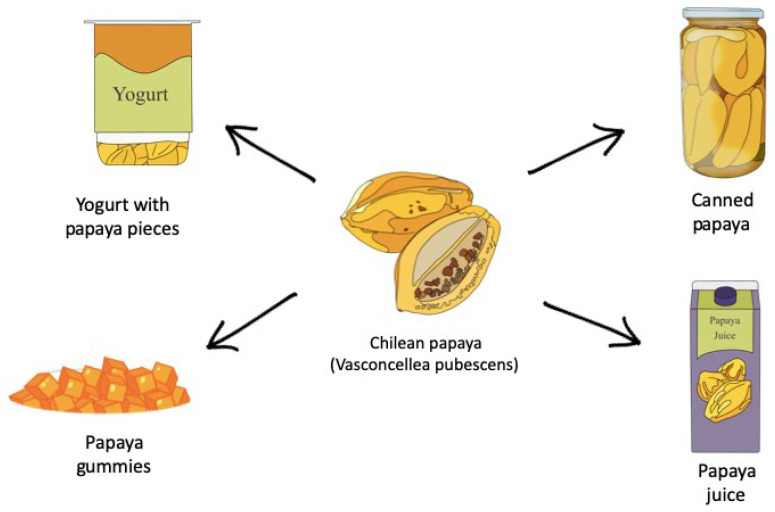
Consumption forms of Chilean papaya.

Papaya is the fruit that reaches the highest rate of industrialization in the national average, between 80 and 90% [95]. Some of the most commercialized products in Chile are observed in Figure 6.

The main challenges in large-scale processing of *Vasconcellea pubescens* focus on strategies to preserve its bioactive compounds (such as polyphenols, vitamin C, and carotenoids), which are highly susceptible to external conditions. This is where gentle methods, such as the encapsulation of Chilean papaya compounds [83,90], and non-thermal processing technologies, such as high-hydrostatic pressure processing [1,2], stand out. Furthermore, optimizing extraction and storage conditions is essential to minimize the degradation of these valuable compounds. However, the processing of Chilean papaya is limited to a small niche, which belongs to a small region of the country. For this reason, it is essential to conduct more studies related to how the processing of this product affects its characteristics.

In recent years, several products and procedures related to *Vasconcellea pubescens* for production, improving genetic and formulation purposes, as well as the extraction and use of the papain enzyme, have also been patented. Many patents focus primarily on methods for extracting, purification, and stabilization, and various health, chemical, and food applications of papain (approximately 4000 patents). This broad array of patents relating to papain predominantly encompasses the varieties *Vasconcellea pubescens* and *Carica papaya*, with a chronological search covering the period from 1910 to 2024 [96]. A brief description of the most relevant patents is presented in the following section (Table 8).

## 13. Conclusions

Due to its production and consumption, Chilean papaya is an essential fruit in Chilean culture. *Vasconcellea pubescens* is rich in vitamins and nutrients and promotes a healthy digestive system thanks to papain enzymes. It also helps prevent cardiovascular diseases and diabetes, owing to its bioactive compounds such as phenols, flavonoids, and carotenoids, which possess antioxidant, anti-inflammatory, and antidiabetic potential, among other health benefits. As shown in this review, papaya latex is a vital enzyme extracted from unripe fruits, with proportions varying depending on the size of the papaya but remaining consistent regardless of the season. Papain is a proteolytic enzyme present in the latex of the fruit that not only facilitates digestion by breaking down proteins, but also has applications in cosmetics, food products, and wound treatments due to its regenerative effects. Additionally, this enzyme can inhibit α-glucosidase, offering the potential for controlling type II diabetes.

Chilean papaya also has a variety of processing methods that achieve different results in its nutritional composition, such as levels of antioxidants and phenolic compounds. Encapsulation technologies, such as alginate and maltodextrin, have been proven effective in protecting and releasing bioactive compounds under controlled conditions, opening possibilities for developing supplements and nutraceuticals. Furthermore, innovations in extraction processes, such as HHP and US, optimize the recovery of antioxidant and antimicrobial compounds. These advances could position Chilean papaya as a high-value functional product in international markets.

The main differences between papaya species (*Vasconcellea*) are their geographical distribution, morphology (size), resistance to environmental conditions, and papain content. Regarding the latter, papain content is higher in Andean species like *V. pubescens*, making it an essential molecule for the food industry. Finally, research on Chilean papaya must continue exploring methods to improve, scale up, patent, and optimize its production process, extend its shelf life and availability, and incorporate it more broadly into people’s diets.

## Figures and Tables

**Table 1:** Different types of *Caricacea*, based on genera, species, and origin.

Genera	Species	Origin	Image	References
*Carica*	*C. papaya*	Guatemala Ecuador	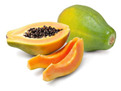 * C. papaya *	[12]
*Cylicomorpha*	*C. parviflora* *C. solmsii*	Tanzania Cameroon	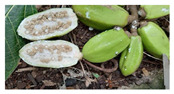 *C. parviflora*	[13]
*Vasconcellea*	*V. candicans* *V. cauliflora* *V. crassipetala* *V. glandulosa* *V. goudotiana* *V. horovitziana* *V. longiflora* *V. microcarpa* *V. monoica* *V. omnilingua* *V. palandensis* *V. parviflora* *V. pubescens* *V. pulchra* *V. quercifolia* *V. sphaerocarpa* *V. sprucei* *V. stipulata* *V. weberbaueri* *V. chilensis* *V. x heilbornii* (*hybrid*)	Peru Guatemala Ecuador Argentina Colombia Bolivia Chile	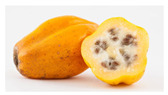 *V. pubescens*	[14]
*Jacaratia*	*J. digitata* *J*, *spinosa* *J. chocoensis* *J. corumbensis* *J. dolichaula* *J. mexicana* *J. heptaphylla*	Ecuador Peru Paraguay Mexico Brazil	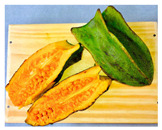 *J. mexicana*	[15]
*Horovitzia*	*H. cnidoscoloides*	Mexico	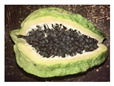 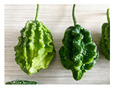 *H. cnidoscoloides*	[16,17]
*Jarilla*	*J. chocola* *J. caudata* *J. heterophylla*	Guatemala	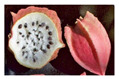 *J. chocola*	[18]

**Table 2:** Comparison between *V. pubescens* and *V. chilensis*.

Sections	*Vasconcellea pubescens*	*Vasconcellea chilensis*	References
Fruit	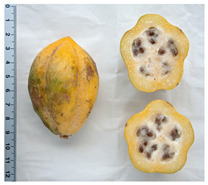	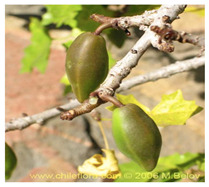	[34,35]
Leaves	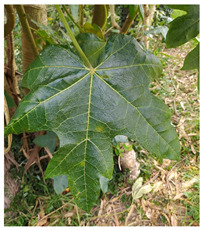	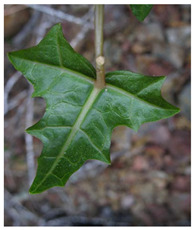	[36,37]
Flowers	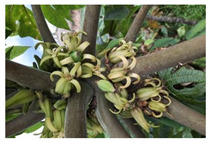	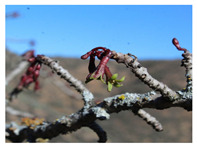	[38,39]
Tree (bush/plant)	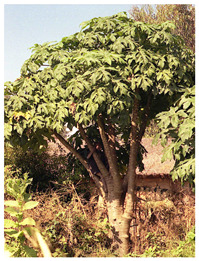	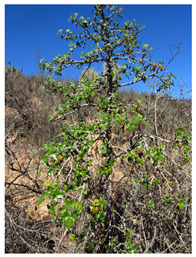	[40,41]
Seed	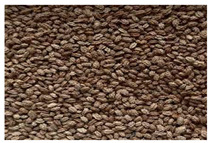	No photo	[42]

**Table 3:** Nutritional and proximal composition of fresh papaya.

Reference	[50]	[1]	[51]
Component	*Vasconcellea pubescens*		*Carica papaya*
Energy (kcal)	18	N/A	N/A
Protein (g)	1.0	0.9	1.17
Total lipid (g)	0.3	0.3	0.49
Carbohydrate (g)	N/A	4.9	9.51
Total dietary fiber (g)	1.4	1.1	0.83
Moisture	93.2	91.6	87.47
Ash	0.8	0.6	0.53
pH	N/A	4.1	N/A
Acidity (%)	N/A	0.1	N/A
Calcium (mg)	36	N/A	30.73
Iron (mg)	1.3	N/A	2.31
Magnesium (mg)	N/A	N/A	12.80
Phosphorus (mg)	28	N/A	29.80

N/A: Not applicable. Schmidt Hebbel et al. (1990) [50]; Nwofia et al. (2012) [51]; and Uribe et al. (2015) [1].

**Table 5:** Latex component and enzyme activity of *Vasconcellea pubescens*.

Reference	[59]	[60]	[64]	[65]
Component	Processes			
	Crude Extract	Freeze-Drying	Hot-Air Drying (70 °C)	pH 6.0
Solid matter (%)	15	20.7	N/A	15.93
Enzyme (%)	40	N/A	N/A	34.20
Enzyme activity	N/A	24.13 U mg^−1^ enzyme	29.9 U mg^−1^ enzyme	195.80 Upe
After processes	15	20.7	N/A	15.93

N/A: Not applicable. Chen et al. (2012) [59]; Vidal et al. (2009) [60]; Jiménez et al. (2023) [64]; Rivera-Botonares et al. (2023) [65].

**Table 6:** Comparative of total phenolic, total flavonoid, and DPPH radical-scavening of three fruits.

Reference	[48]	[78]	[10]	[79]
Extraction	Pulp	Pulp		Fruit
	*Vasconcellea* *pubescens*	*Carica papaya* L.		* Nauclea latifolia *
TPC ^2^	7.02 ± 0.42 mg GAE g^−1^ D.M	30.7 ± 2.7 mg GAE g^−1^ D.M.	N/A	44.56 ± 0.78 mg GAE g^−1^
TFC ^3^	3.33 ± 0.26 mg QE g^−1^ D.M.	19.1 ± 1.5 mg CE g^−1^ D.M.	N/A	N/A
DPPH ^4^	81.26 ± 1.23 µmol TE g^−1^ D.M.	N/A	4.41 ± 0.28 mmol TE 100 g^−1^	N/A
IC_50_	312 mg mL^−1^	N/A	1.58 ± 0.26 mg mL^−1^	6.94 µg mL^−1^

N/A: Not applicable, ^2^ TPC: total phenolic content, ^3^ TFC: total flavonoid content, ^4^ DPPH: 2,2-diphenyl-1-picrylhydrazyl. Vega-Gálvez et al. (2019) [48]; Mishra et al. (2015) [78]; Mohamed et al. (2024) [79].

**Table 7:** Different processes of Chilean papaya.

Method	Material Condition	Treatment Condition	Bioactive Compounds Results	Reference
Agitation Extraction	Fruit with skin, 5 g homogenized	Aqueous methanol: 80% Solid/liquid: 1:4 Agitation: 200 rpm Time: 30 min	Fraction free ^1^ TPC: 23.8 mg GAE g^−1^ D.M. ^3^ DPPH: 17.6 mM TE 100 g^−1^ ^5^ FRAP: 100 mM TE 100 g^−1^ Voltammetry: 15.2 mM TE 100 g^−1^ Rutin: 2.0 mg 100 g Fraction bound ^5^ FRAP: 28.1 mM TE 100 g^−1^ Voltammetry: 2.1 mM TE 100 g^−1^ *p*-Coumaric acid: 0.1 mg 100 g^−1^	[1]
Convective Drying	Slices 9.0 × 1.5 × 0.4 cm (length × width × thickness)	Temperature: 70 °C Time: 270 min Air velocity: 1.5 m s^−1^	^1^ TPC: 7.07 mg GAE g^−1^ D.M. ^2^ TFC: 1.25 mg QE g^−1^ D.M. ^3^ DPPH: 27.78 μmol TE g^−1^ D.M. ^4^ ORAC: 75.47 μmol TE g^−1^ D.M. β-Carotene: 1762 µg 100 g^−1^ D.M Vitamin C: 4.07 mg g^−1^ D.M Gallic acid: 5.66 mg 100 g^−1^ D.M Chlorogenic acid: 3.66 mg 100 g^−1^ D.M Tyrosol: 20.16 mg 100 g^−1^ D.M Naringin: 2.01 mg 100 g^−1^ D.M. *p*-Coumaric acid: 2.52 mg 100 g^−1^ D.M. Trans-ferulic acid: 1.98 mg 100 g^−1^ D.M.	[48]
Convective Drying	Papaya pulp with a peel Initial moisture: 92.72 g 100 g^−1^	Temperature: 60 °C Time: 5.5 h Air velocity: 60% Relative humidity: 65–73%	^1^ TPC: 5.19 mg GAE g^−1^ D.M. ^2^ TFC: 2.00 mg QE g^−1^ D.M. ^3^ DPPH: 15.13 μmol TE g^−1^ D.M. ^4^ ORAC: 49.31 μmol TE g^−1^ D.M.	[55]
Freeze-drying	Papaya pulp with a peel Initial moisture: 92.72 g 100 g^−1^	Freezing: −80 °C Vacuum: 0.027 kPa. Time: 73 h.	^1^ TPC: 4.82 mg GAE g^−1^ D.M. ^2^ TFC: 2.37 mg QE g^−1^ D.M. ^3^ DPPH: 21.16 μmol TE g^−1^ D.M. ^4^ ORAC: 35.24 μmol TE g^−1^ D.M.	[55]
Freeze-drying	Slices 9.0 × 1.5 × 0.4 cm (length × width × thickness) Freezing: −80 °C. Time: 24 h	Ramps temperature: − 40 to 15 °C Vacuum: 0.027 kPa Time: 73 h	^1^ TPC: 6.76 mg GAE g^−1^ D.M. ^2^ TFC: 1.17 mg QE g^−1^ D.M. ^3^ DPPH: 29.12 μmol TE g^−1^ D.M. ^4^ ORAC: 45.92 μmol TE g^−1^ D.M. β-Carotene: 1471 µg 100 g^−1^ D.M. Vitamin C: 4.95 mg g^−1^ D.M. Gallic acid: 5.01 mg 100 g^−1^ D.M. Chlorogenic acid: 2.90 mg 100 g^−1^ D.M. Tyrosol: 8.55 mg 100 g^−1^ D.M. Naringin: 3.05 mg 100 g^−1^ D.M. *p*-Coumaric acid: 1.67 mg 100 g^−1^ D.M. Trans-ferulic acid: 1.21 mg 100 g^−1^ D.M.	[48]
HHP Extraction	Fruit with skin, 5 g homogenized	Pressure: 500 MPa Time: 10 min	Fraction free ^1^ TPC: 28.6 mg GAE g^−1^ D.M. ^3^ DPPH: 16.2 mM TE 100 g^−1^ ^5^ FRAP: 101.9 mM TE 100 g^−1^ Voltammetry: 16.9 mM TE 100 g^−1^ Rutin: 1.9 mg 100 g^−1^ Fraction bound ^5^ FRAP: 32.3 mM TE 100 g^−1^ Voltammetry: 1.6 mM TE 100 g^−1^ *p*-Coumaric acid: 0.2 mg 100 g^−1^ *trans*-Ferulic acid: 0.2 mg 100 g^−1^	[1]
HHP Extraction	Seeds washed Air dried in the dark and stored at 18 °C	Pressure: 500 MPa Time: 5, 10 and 15 min Pulses: 1 min	Efficiency extraction methods ^1^ TPC: 70.0% × 5 min, 92.1% ×10 min, 111.7% × 15 min ^2^ TFC: 89.70% × 5 min, 166.1% × 10 min, 277.0% × 15 min ^3^ DPPH: 129.3% × 5 min, 242.7% × 10 min, 272.8% × 15 min ^5^ FRAP: 176.7% × 5 min, 193.1% × 10 min, 269.3% × 15 min Sulforaphane content: 41.44 (5 min), 57.48 (10 min), 54.97 (15 min) mg g^−1^ seed	[2]
HHP Agitation Extraction	Fruit with skin, 5 g homogenized	Pressure: 500 MPa Time: 5 min Agitation: 200 rpm Time: 15 min	Fraction free ^1^ TPC: 126.9 mg GAE g^−1^ D.M. ^3^ DPPH: 20.5 mM TE 100 g^−1^ ^5^ FRAP: 101.1 mM TE 100 g^−1^ Voltammetry: 140.5 mM TE 100 g^−1^ Caffeic acid: 1.6 mg 100 g^−1^ *trans*-Ferulic acid: 0.82 mg 100 g^−1^ Rutin: 2.8 mg 100 g^−1^ Fraction bound ^1^ TPC: 0.9 mg GAE g^−1^ D.M. ^3^ DPPH: 2.1 mM TE 100 g^−1^ ^5^ FRAP: 83.4 mM TE 100 g^−1^ Voltammetry: 59.4 mM TE 100 g^−1^ *p*-Coumaric acid: 0.6 mg 100 g^−1^ *trans*-Ferulic acid: 0.5 mg 100 g^−1^	[1]
HHP-Ultrasound Extraction	Fruit with skin, 5 g homogenized	Pressure: 500 MPa Time: 5 min Ultrasound: 60 Hz Time: 15 min	Fraction free ^1^ TPC: 129.1 mg GAE g^−1^ D.M. ^3^ DPPH: 20.6 mM TE 100 g^−1^ ^5^ FRAP: 97.2 mM TE 100 g^−1^ Voltammetry: 141.0 mM TE 100 g^−1^ Caffeic acid: 1.5 mg 100 g^−1^ *trans*-Ferulic acid: 0.86 mg 100 g^−1^ Rutin: 2.8 mg 100 g^−1^ Fraction bound ^1^ TPC: 1.2 mg GAE g^−1^ D.M. ^3^ DPPH: 1.8 mM TE 100 g^−1^ ^5^ FRAP: 85.7 mM TE 100 g^−1^ Voltammetry: 27.7 mM TE 100 g^−1^ *p*-Coumaric acid: 0.4 mg 100 g^−1^ *trans*-Ferulic acid: 0.3 mg 100 g^−1^	[1]
Hot Air Drying and Osmotic Pretreatment	Slabs of 10.0 mm	Temperature (°C) Sucrose solutions (%*w*/*w*) Treatments (T_n = 1–6_) T_1_: 40 °C T_2_: 60 °C T_3_: 80 °C T_4_: 60 °C-40% *w*/*w* T_5_: 60 °C-50% *w*/*w* T_6_: 60 °C-60% *w*/*w*	Percentage of Vitamin C decrease T_1_: 77.21% T_2_: 74.41% T_3_: 81.75% T_4_: 83.05% T_5_: 81.60% T_6_: 82.58%	[93]
Infrared Drying	Slices 9.0 × 1.5 × 0.4 cm (length × width × thickness)	Temperature: 70 °C Time: 390 min Lamp IR: 175 W	^1^ TPC: 7.11 mg GAE g^−1^ D.M. ^2^ TFC: 1.93 mg QE g^−1^ D.M. ^3^ DPPH: 25.64 μmol TE g^−1^ D.M. ^4^ ORAC: 96.26 μmol TE g^−1^ D.M. β-Carotene: 1544 µg 100 g^−1^ D.M. Vitamin C: 3.48 mg g^−1^ D.M. Gallic acid: 8.65 mg 100 g^−1^ D.M. Chlorogenic acid: 4.21 mg 100 g^−1^ D.M. Tyrosol: 16.10 mg 100 g^−1^ D.M. Naringin: 1.49 mg 100 g^−1^ D.M. *p*-Coumaric acid: 7.84 mg 100 g^−1^ D.M. Trans-ferulic acid: 5.56 mg 100 g^−1^ D.M.	[48]
Infrared Drying	Papaya pulp with peel Initial moisture: 92.72 g 100 g^−1^	Temperature: 60 °C Time: 7 h Lamp IR: 175 W	^1^ TPC: 4.29 mg GAE g^−1^ D.M. ^2^ TFC: 2.29 mg QE g^−1^ D.M. ^3^ DPPH: 11.45 μmol TE g^−1^ D.M. ^4^ ORAC: 66.83 μmol TE g^−1^ D.M.	[55]
Low Temperature Vacuum Drying	Papaya pulp Initial moisture: 92%	Temperature: 20 °C Time: 38 h Vacuum: 1 kPa	^1^ TPC: 4.66 mg GAE g^−1^ D.M. ^2^ TFC: 1.53 mg QE g^−1^ D.M. ^3^ DPPH: 20.80 μmol TE g^−1^ D.M. ^4^ ORAC: 46.69 μmol TE g^−1^ D.M.	[55]
Solar Drying	Slices 9.0 × 1.5 × 0.4 cm (length × width × thickness)	Temperature: 31.0–49.9 °C Relative humidity: 20–45% Time: 870 min	^1^ TPC: 6.45 mg GAE g^−1^ D.M. ^2^ TFC: 1.36 mg QE g^−1^ D.M. ^3^ DPPH: 25.89 μmol TE g^−1^ D.M. ^4^ ORAC: 86.56 μmol TE g^−1^ D.M. β-Carotene: 700 µg 100 g^−1^ D.M. Vitamin C: 2.14 mg g^−1^ D.M. Gallic acid: 2.93 mg 100 g^−1^ D.M. Chlorogenic acid: 1.97 mg 100 g^−1^ D.M. Tyrosol: 9.40 mg 100 g^−1^ D.M. Naringin: 0.76 mg 100 g^−1^ D.M. *p*-Coumaric acid: 1.67 mg 100 g^−1^ D.M. Trans-ferulic acid: 2.12 mg 100 g^−1^ D.M.	[48]
Ultrasound Extraction	Fruit with skin, 5 g homogenized	Aqueous methanol: 80% Solid/liquid: 1:4 Ultrasound: 60 Hz Time: 30 min	Fraction free ^1^ TPC: 26.3 (mg GAE g^−1^ D.M. ^3^ DPPH: 15.7 mM TE 100 g^−1^ ^5^ FRAP: 99.9 mM TE 100 g^−1^ Voltammetry: 12.9 mM TE 100 g^−1^ Rutin: 2.0 mg 100 g^−1^ Fraction bound ^5^ FRAP: 22.9 mM TE 100 g^−1^ Voltammetry: 3.3 mM TE 100 g^−1^ *p*-Coumaric acid: 2.0 mg 100 g^−1^	[1]
Ultrasound-Assisted Extraction	Seeds washed Air dried in the dark and stored at 18 °C	Solid/liquid Ratio: 1:2 Time: 5, 10 and 15 min	Efficiency extraction methods ^1^ TPC: 3.9% × 5 min, 13.1% × 10 min, 19.2% × 15 min ^2^ TFC: 10.5% × 5 min, 26.28% × 10 min, 51.0% × 15 min ^3^ DPPH: 9.3% × 5 min, 40.4% × 10 min, 66.8% × 15 min ^5^ FRAP: 5.0% × 5 min, 22.4% × 10 min, 45.5% × 15 min Sulforaphane content: 32.32 (5 min) mg g^−1^ seed, 38.81 (10 min) mg g^−1^ seed, 47.82 (15 min) mg g^−1^ seed	[2]
Vacuum drying	Fruit pulp with peel	Vacuum: 15 kPa. Temperature: 60 °C Time: 8 h	^1^ TPC: 5.83 mg GAE g^−1^ D.M. ^2^ TFC: 2.50 mg QE g^−1^ D.M. ^3^ DPPH: 18.26 μmol TE g^−1^ D.M. ^4^ ORAC: 54.38 μmol TE g^−1^ D.M.	[55]
Vacuum drying	Slices 9.0 × 1.5 × 0.4 cm (length × width × thickness)	Temperature: 70 °C Pressure: 15 kPa Time: 480 min	^1^ TPC: 8.89 mg GAE g^−1^ D.M. ^2^ TFC: 1.31 mg QE g^−1^ D.M. ^3^ DPPH: 34.51 μmol TE g^−1^ D.M. ^4^ ORAC: 107.1 μmol TE g^−1^ D.M. β-Carotene: 1469 µg 100 g^−1^ D.M. Vitamin C: 5.39 mg g^−1^ D.M. Gallic acid: 9.76 mg 100 g^−1^ D.M. Chlorogenic acid: 4.38 mg 100 g^−1^ D.M. Tyrosol: 21.01 mg 100 g^−1^ D.M. Naringin: 3.34 mg 100 g^−1^ D.M. *p*-Coumaric acid: 3.97 mg 100 g^−1^ D.M. Trans-ferulic acid: 2.56 mg 100 g^−1^ D.M.	[48]

^1^ Total phenolic content (TPC); ^2^ Total flavonoid content (TFC); ^3^ 2,2-diphenyl-1-picrylhydrazyl (DPPH); ^4^ radical absorbance capacity (ORAC); ^5^ ferric-reducing antioxidant power (FRAP).

**Table 8:** Patent title, code, and classifications for *Vasconcellea pubescens*.

Title	Code	Product	Classification
Procedure for the preparation of a vegetable rennet based on the lyophilized papain enzyme extracted from native species of the genus *Vasconcellea*	PE2024-0644	Freeze-dried papain enzyme	Food vegetal production
Use of pharmaceutical composition in the preparation of medicines for the treatment of ocular wounds	BR102018015770	Protein fraction P1G10	Pharmaceutical formulation
Stabilized proteases for use in skin care	ES2596402	Stabilized papain enzyme	Topical skin applications
Antimicrobial formulation comprising metal nanoparticles or metal oxides synthesized from plant extracts	WO2022168070	Plant extracts (leaves, stems, seeds, flowers, fruits, latex, roots or peels)	Biocidal from plant extracts
In-vitro culture method of plant tissues by direct organogenesis of the babaco hybrid [*Vasconcellea* Ã Heilbornii (Badillo) Badillo]	ECSP21088416	Improved genetically fruit	Genetic modification in fruits and vegetables

## Data Availability

The data presented in this study are available on request from the corresponding author.
